# Biomechanical evaluation of a newly designed locking plate for opening wedge high tibial osteotomy: stress distribution and stability in the presence of lateral hinge fracture

**DOI:** 10.1186/s13018-024-05283-w

**Published:** 2024-11-27

**Authors:** Hyung Jun Park, Dong Hun Suh, Hyongtaek Hong, Kyung-Wook Nha, Hyungsuh Kim, Kyoung-Tak Kang, Jae Gyoon Kim

**Affiliations:** 1 Department of Orthopedic Surgery, Korea University Ansan Hospital, Korea University College of Medicine, Ansan-Si, Gyeongki-Do Republic of Korea; 2 Skyve R&D Lab, Seoul, Republic of Korea; 3 Department of Orthopedic Surgery, Ilsanpaik Hospital, Inje University College of Medicine, Goyangsi, Republic of Korea; 4 Department of Mechanical Engineering, Yonsei University, Seoul, Republic of Korea; 5 Skyve R&D Lab, 11, Dangsan-Ro 41-Gil, Yeongdeungpo-Gu, Seoul, Republic of Korea; 6 Department of Mechanical Engineering, Yonsei University, 50, Yonsei-Ro, Seodaemun-Gu, Seoul, Republic of Korea; 7 Department of Orthopedic Surgery, Korea University Ansan Hospital, Korea University College of Medicine, 123, Jeokgeum-Ro, Danwon-Gu, Ansan-Si, Gyeongki-Do 425-707 Republic of Korea

**Keywords:** Opening wedge high tibial osteotomy, Lateral hinge fracture, Locking plate biomechanics, Stress distribution

## Abstract

**Background:**

The study aimed to evaluate whether a new OhtoFix plate reduced stress around the D-hole compared with an old OhtoFix and TomoFix plate. The study also assessed whether the new OhtoFix plate had biomechanical stability in a lateral hinge fracture (LHF).

**Methods:**

A finite element model of the proximal tibia was developed using cross-sectional images from a 62-year-old Asian woman. The model was designed to simulate opening wedge high tibial osteotomy (OWHTO) using three types of locking plates: the newly designed locking plate (new OhtoFix plate), the prior version of the OhtoFix plate (old OhtoFix plate), and the TomoFix plate. The peak von Mises stress (PVMS) was analyzed around the D-hole and across the entire plate including the impact of different LHF types classified according to the Takeuchi classification.

**Results:**

The new OhtoFix plate significantly reduced stress around the D-hole compared with the old OhtoFix and TomoFix plates, with peak stresses of 189.5 MPa, 251.5 MPa, and 233.3 MPa, respectively. Despite this improvement, the new OhtoFix plate did not surpass the TomoFix in terms of overall stress distribution across the entire plate. Additionally, in cases of LHF, although peak stress remained at the D-hole in both the old OhtoFix and TomoFix plates, the peak stress shifted to the C-hole in the new plate.

**Conclusions:**

The new OhtoFix plate improved stress distribution around the D-hole (even in the presence of a LHF) compared to the old OhtoFix plate and TomoFix plates. However, although the new OhtoFix plate reduced peak stress around the D-hole, it did not demonstrate superior overall stress distribution across the entire plate compared to the TomoFix plate.

## Background

Stable fixation around the osteotomy site is critical for preventing adverse outcomes in medial opening wedge high tibial osteotomy (OWHTO) [1]. An open osteotomy in the proximal tibia induces an unstable condition necessitating a fixation strategy that ensures structural stability to facilitate optimal bone healing [2]. Inadequate fixation can lead to correction loss, non-union, and early need for total knee arthroplasty [2, 3]. Over the years, various plates have been developed and utilized for stable fixation; however, angular stable locking plates have emerged as the most reliable option for achieving secure fixation [2, 4, 5]. Although a variety of locking plate designs are available, no consensus exists regarding which locking plate demonstrates biomechanical superiority [2, 5–7].

Moreover, despite the use of locking plates, fixation failure around the osteotomy site may still occur in OWHTO [8]. One study reported breakage of locking plates around the D-hole [9]. That study reported that the risk of plate breakage increased in the presence of a lateral hinge fracture (LHF), irrespective of the type of locking plate used in OWHTO [9]. The incidence of LHF in OWHTO has been reported to range from 19.8 to 41.2%, with the risk increasing in cases requiring a larger correction angle [10–15]. When LHF occurs during OWHTO, instability at the osteotomy site increases, leading to loss of correction, with or without delayed union [12, 16, 17]. These complications have been reported to be more frequent in LHF Takeuchi types II and III [18]. In response to the risk of plate breakage, a newly designed OhtoFix plate (Ohtomecal, Goyang-si, Gyeonggi-do, Republic of Korea), with increased thickness and width around the D-hole, was developed and has been widely used by Korean orthopedic surgeons [19]. Additionally, the TomoFix plate was modified by eliminating the combi hole at the D-hole, retaining only the locking screw hole [20]. However, the biomechanical stability of this newly designed locking plate has not yet been evaluated.

We aimed to evaluate whether the newly designed locking plate effectively reduces stress around the D-hole and across the entire plate compared with the existing plates. Additionally, we assessed whether the newly designed locking plate maintains its biomechanical stability in the presence of LHF. We hypothesized that the newly designed locking plate would demonstrate reduced stress and maintain its stability even in the event of LHF.

## Methods

### Development of normal proximal tibia finite element model

Cross-sectional images of the lower extremities of a normal adult, a 62-year-old Asian woman, were obtained using a 64-channel computed tomography scanner (Somatom Sensation 64; Siemens Healthcare, Erlangen, Germany) with a slice interval of 0.1 mm. A three-dimensional geometric contour of the tibia was generated by stacking the CT cross-sectional images using the Mimics program (version 21.0, Materialise Inc., Belgium). The bone surfaces were then refined into more sophisticated solid models using the Unigraphics NX program (version 7.0; Siemens PLM Software, Torrance, CA). A finite element mesh was created using the HyperMesh program (version 8.0; Altair Engineering, Troy, MI). The study protocol was approved by an institutional review board (18-DR-02).

### Development of proximal tibia FE model with plate removal after OWHTO

#### Development of the OWHTO model

**Fig. 1:**
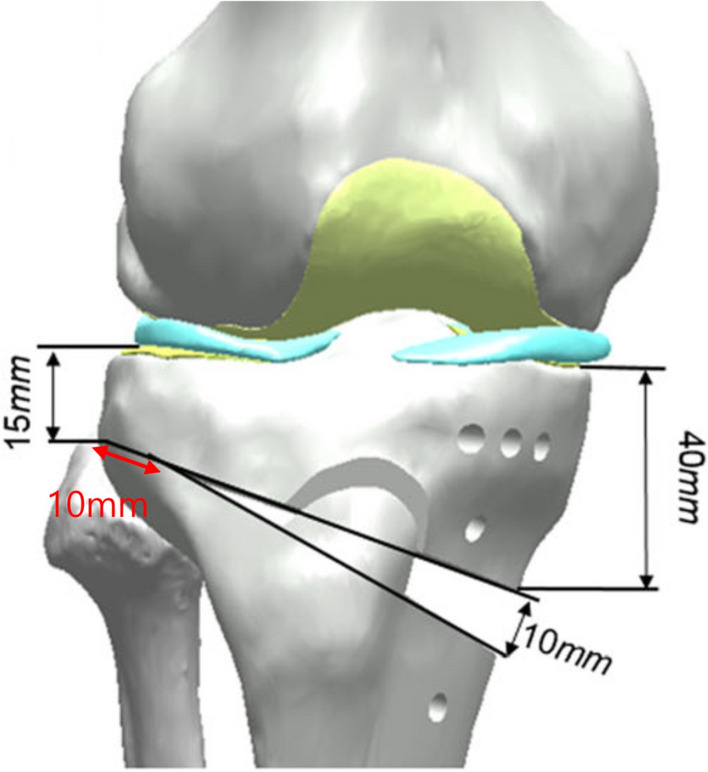
Schematic illustrating opening wedge high tibial osteotomy model

**Fig. 2:**
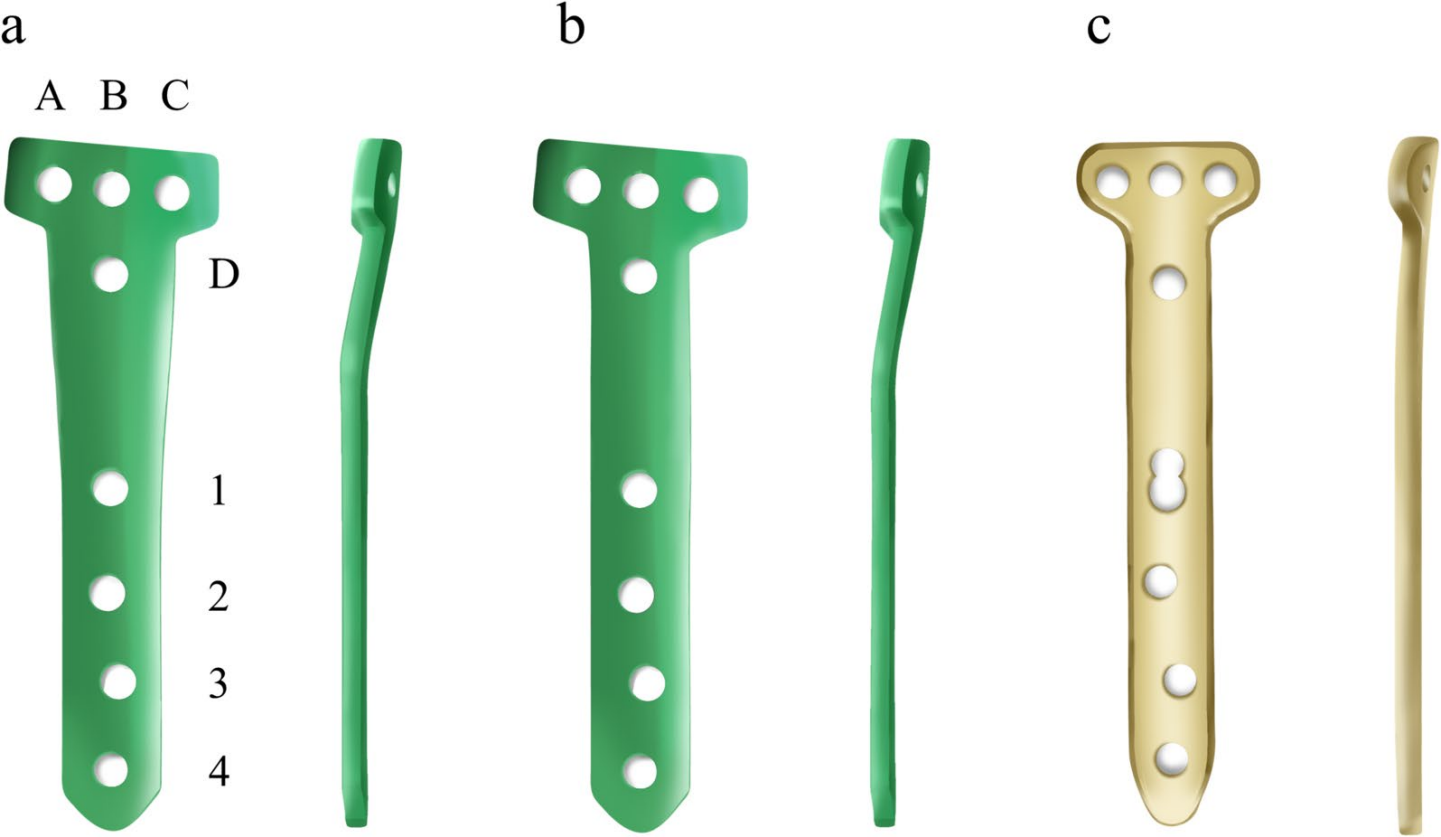
Comparison of locking plate designs; **b** New OhtoFix; **c** Old OhtoFix; **d** TomoFix Plate

**Fig. 3:**
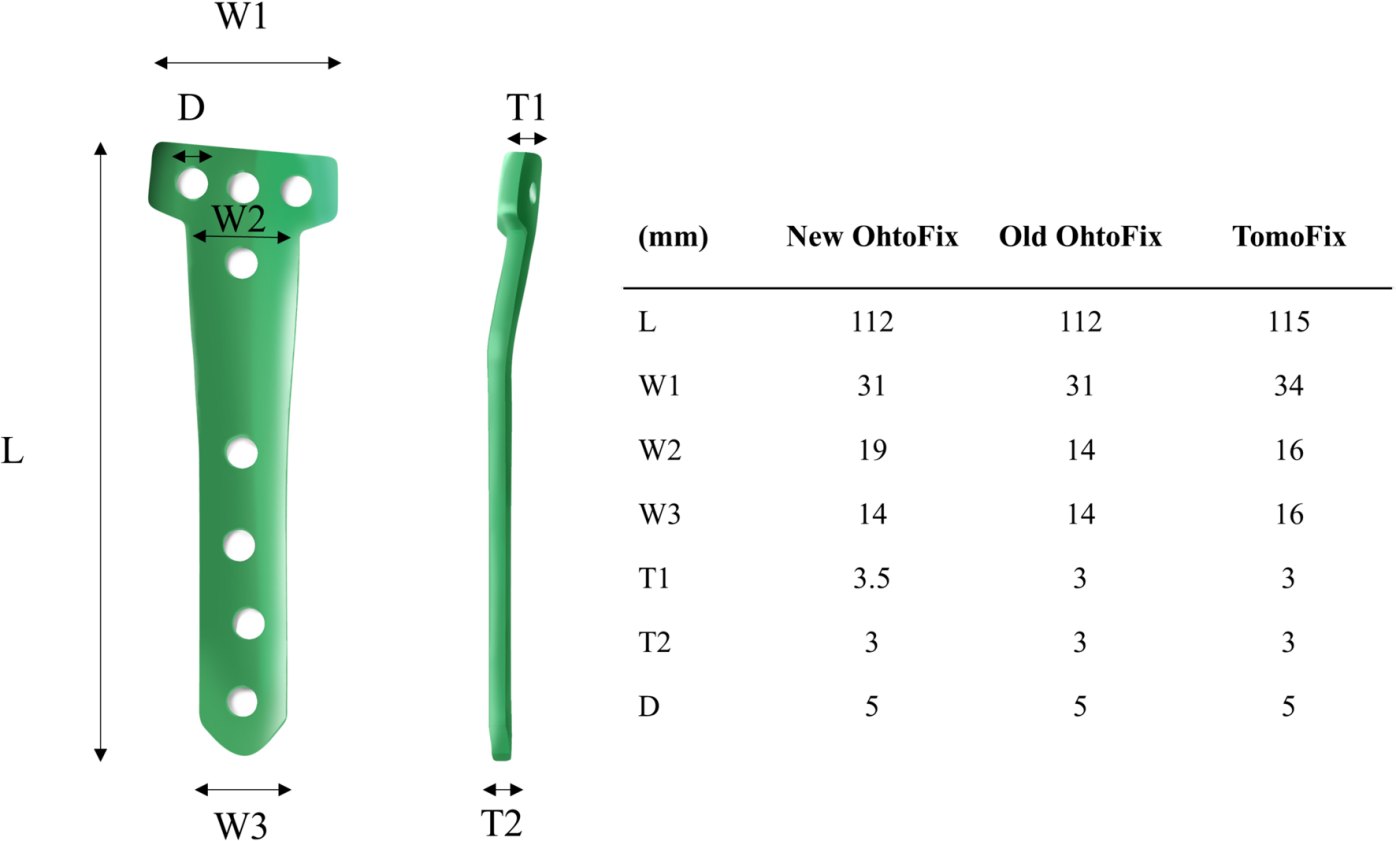
Dimensions and specifications of the New OhtoFix, Old OhtoFix, and TomoFix Plates: detailed measurements of length, thickness, and the name of each hole

**Fig. 4:**
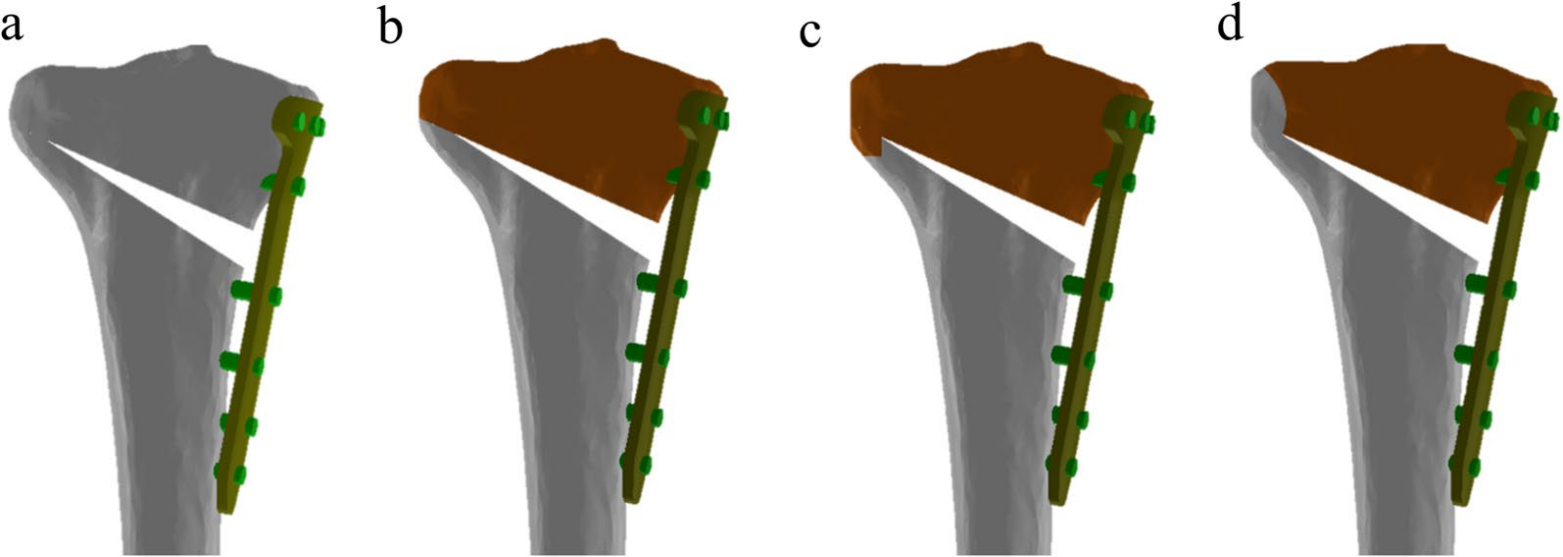
Finite element models used in this study **a** opening wedge high tibial osteotomy without lateral hinge fracture; **b** Takeuchi classification for lateral hinge fracture type I; **c** type II; **d** type III

The constructed FE model was subsequently used to simulate OWHTO with the distal region of the tibia rotated, while the opening wedge was simulated in the frontal plane to achieve the desired valgus correction angle [21]. The opening wedge on the medial side was guided by a clinician to represent OWHTO. Wedge size and correction angle were determined based on previous studies [21, 22]. An OWHTO model with a 10 mm opening gap was created to simulate a weight-bearing line passing through 62.5% of the tibia plateau by removing a single-plain wedge-shaped osteotomy bone at the proximal tibia, preserving a 10 mm hinge from the lateral cortex (Fig. 1). For the scope of this study, only the proximal tibia was included in the analysis. Three commercially used locking plates were used in the study: the TomoFix plate (DePuy Synthes, Warsaw, IN, USA), and the new and old versions of the OhtoFix plates. Although the TomoFix plate offers biomechanically stable fixation, the size and length of the plate could cause issues such as local irritation and inadequate fit in Asian patients [5]. To address these challenges, the OhtoFix plate was developed as a low-profile, anatomically shaped locking plate for OWHTO [5]. The three plates were three-dimensionally modeled using the computer-aided design software, SolidWorks (version 2023 SP5.0; Dassault Systèmes, Yvelines Vélizy-Villacoublay, France) (Figs. 2, 3). The plate models were virtually implanted into the proximal medial tibia to simulate OWHTO fixation, then removed to create the proximal tibia model at opening wedge and screw holes. LHF models were generated for each plate model to investigate the impact of LHF, and the fractures were classified into Type I, II, and III according to the Takeuchi classification system (Fig. 4) [17]. To compare variations in stress, parameters of strain and micromotion associated with bone formation were analyzed. First, the peak von Mises stress around the locking plates was compared to the yield strength of the material to evaluate stability. The yield strength value of tibia cortical bone (177.2 MPa for axial compression) was used as a reference from a previous study [23]. Second, the average von Mises stresses on the tibia were evaluated to assess load distribution and the effects of stress shielding. A FE analysis was conducted using the ABAQUS software (version 6.14; Dassault Systemes, France).

#### Material property

The material properties of cortical bone, cancellous bone, locking plate, and screws were obtained from previous studies [24–26]. Cortical and cancellous bone in the tibia were both modeled as linear elastic, isotropic, and homogeneous materials. The Young’s moduli of cortical and cancellous bones were 17,000 MPa and 910 MPa, respectively, and Poisson’s ratios were 0.33 and 0.2, respectively [24, 25]. The locking plate and screws were modeled as a Ti6Al5V ELI with E = 110 GPa and v = 0.3 [26] and threads of the locking screw were simplified and applied as cylindrical.

#### Loading and boundary conditions

**Fig. 5:**
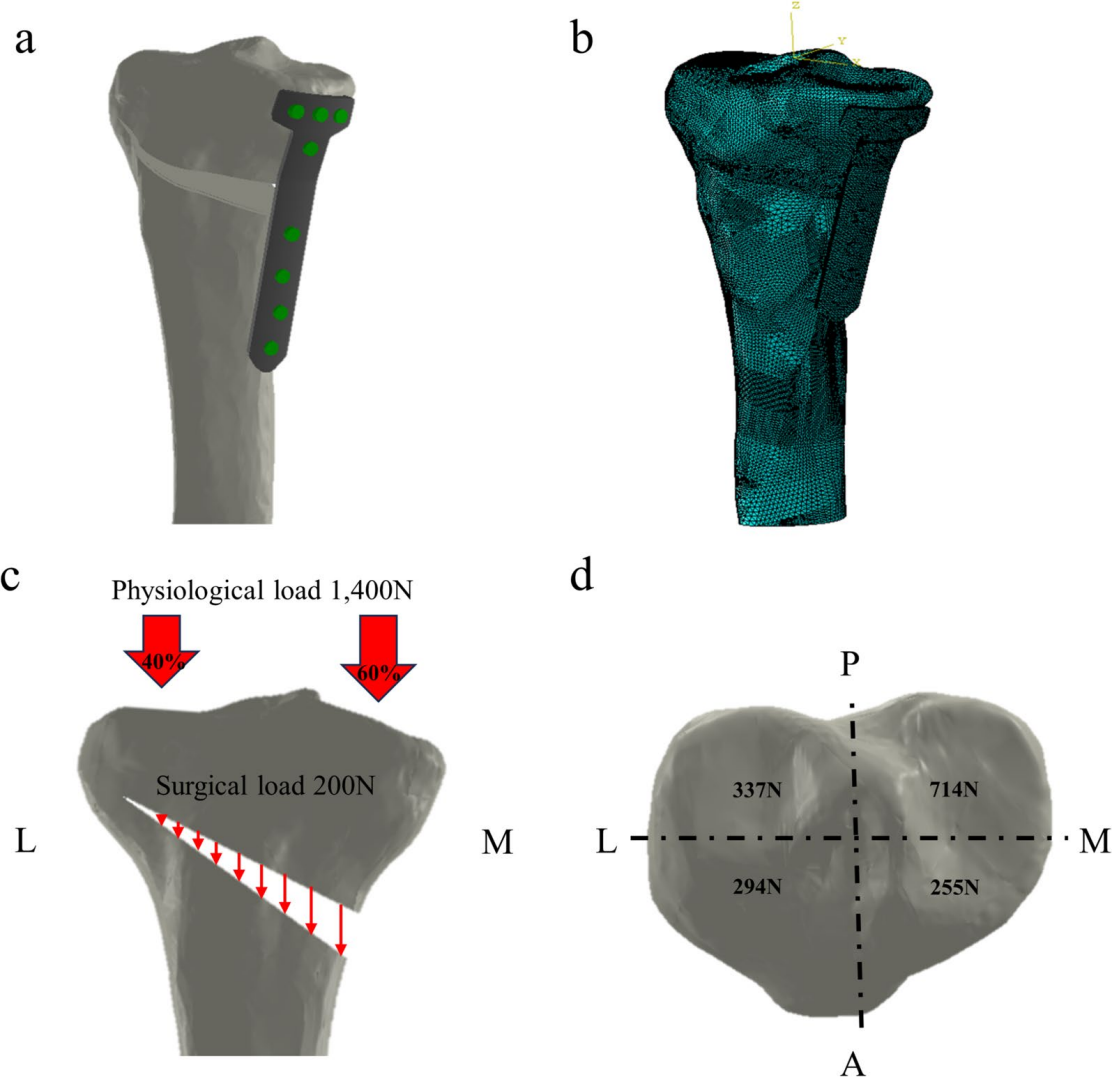
3D model of opening-wedge high tibial osteotomy. **a** solid model, **b** meshed model

Two types of loads were applied to the OWHTO models: intervention-induced compression load and physiological load [24, 27]. The intervention-induced compression load of 200N simulated the stabilizing force consisting of the distraction of the remaining intact cortex, medial collateral ligament, and patellar tendon, which was uniformly applied to the tibia osteotomy site [28]. The physiological load of 1400N was applied to the proximal tibia, with a medial-to-lateral distribution ratio of 60:40 [27]. According to the results from a previous study, restoration of the physiological transfer of the knee load was assumed in this study, and it leads to load repartitions of 40% and 60% on the lateral and medial plateaus, respectively, as shown in Fig. 5c and 5d [27]. A “tie” contact condition was applied assuming full constraints between bone-bone and bone-screws. The distal end of the tibia was assumed to be fully constrained in all tests [29, 30].

### Mesh convergence

Convergence of the FE model was investigated. Mesh convergence was defined as the maximum displacement on trabecular bone within 95% of the pressure of the next two smaller mesh sizes [31]. This criterion was met by a mesh size of 1.0 mm on the locking plate and screws region, 1.0 mm on the stem-around region, and 1.2 mm for other regions. The cortical bone, cancellous bone, plate, and screws have 232 k, 555 k, 30 k, and 46 k elements, and 346 k, 829 k, 46 k, and 75 k nodes, respectively. Quadratic tetrahedral elements of type C3D10 were applied (Fig. 5).

### Intact model validation

The FE model was validated by comparing it with data from a previous study, investigating experimental validation of a finite element model of a human cadaveric tibia [32]. The FE model was validated under torsional loading conditions. The minimum principal strain and maximum principal strain were investigated. The largest values of minimum principal strain on the tibia bone and the reference model were − 542 (micro-strain) and − 569 (micro-strain), respectively. The largest values of maximum principal strain on the tibia bone and the reference model were 403 (micro-strain) and 426 (micro-strain), respectively. The difference was smaller than 10%. Therefore, the FE model used in this study was validated by comparing the differences in the largest values of minimum principal strain and maximum principal strain on the tibia bone between the FE model and literature data.

## Results

**Fig. 6:**
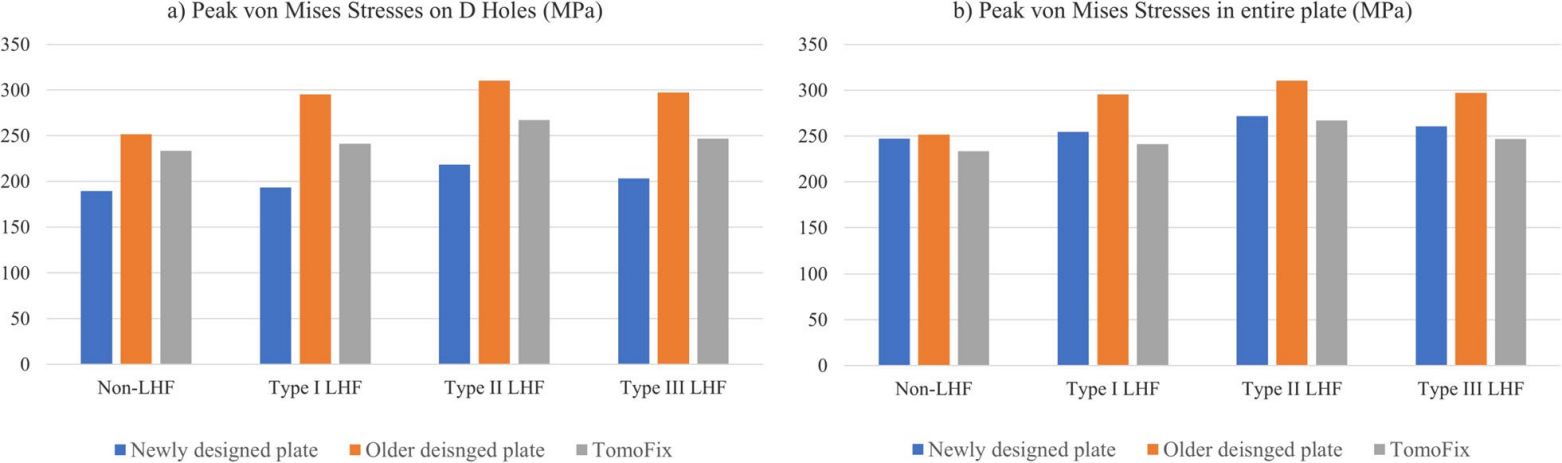
Comparative analysis of peak von Mises stresses (PVMS) under various conditions **a** PVMS around the D-holes; **b** PVMS across the entire plate. A comparison was made among the New OhtoFix, Old OhtoFix, and TomoFix plates in case without lateral hinge fracture, and with Type I, Type II, and Type III lateral hinge fractures

The newly designed locking plate demonstrated lower stress around the D-hole compared to both the old OhtoFix plate and the TomoFix. The peak von Mises stress around the D-hole was 189.5 MPa in the newly designed plate, 251.5 MPa in the old OhtoFix plate, and 233.3 MPa in the TomoFix plate (Table 1, Fig. 6a). Although the newly designed plate showed an improvement in peak von Mises stress across the entire plate compared to the old OhtoFix plate, it did not outperform the TomoFix plate. Additionally, while the location of peak stress remained at the D-hole in both the old OhtoFix and TomoFix plates, it shifted to the 1-hole in the newly designed plate (Fig. 4). The peak von Mises stress across the entire plate was 247.1 MPa at 1-hole in the newly designed plate, compared to 251.5 MPa and 233.3 MPa at the D-hole in the old OhtoFix and in the TomoFix plates, respectively (Table 2, Fig. 6b).

**Table 1:** Peak von Mises stresses around the D-hole in opening-wedge high tibial osteotomy with and without lateral hinge fracture (LHF) among New OhtoFix, Old OhtoFix, and TomoFix plates

	Non-LHF	Type I LHF	Type II LHF	Type III LHF
New OhtoFix	189.5	193.2	218.1	203.2
Old OhtoFix	251.5	295.2	310.4	297.3
TomoFix	233.3	241.1	267.0	246.6

* All values are presented as in MPa

Abbreviation: lateral hinge fracture; LHF

**Table 2:** Peak von Mises stresses around the entire plate in opening-wedge high tibial osteotomy with and without lateral hinge fracture (LHF) among New OhtoFix, Old OhtoFix, and TomoFix plates

	Non-LHF	Type I LHF	Type II LHF	Type III LHF
New OhtoFix	247.1	254.5	271.9	260.6
Old OhtoFix	251.5	295.2	310.4	297.3
TomoFix	233.3	241.1	267.0	246.6

* All values are presented as in MPa

Abbreviation: lateral hinge fracture; LHF

**Fig. 7:**
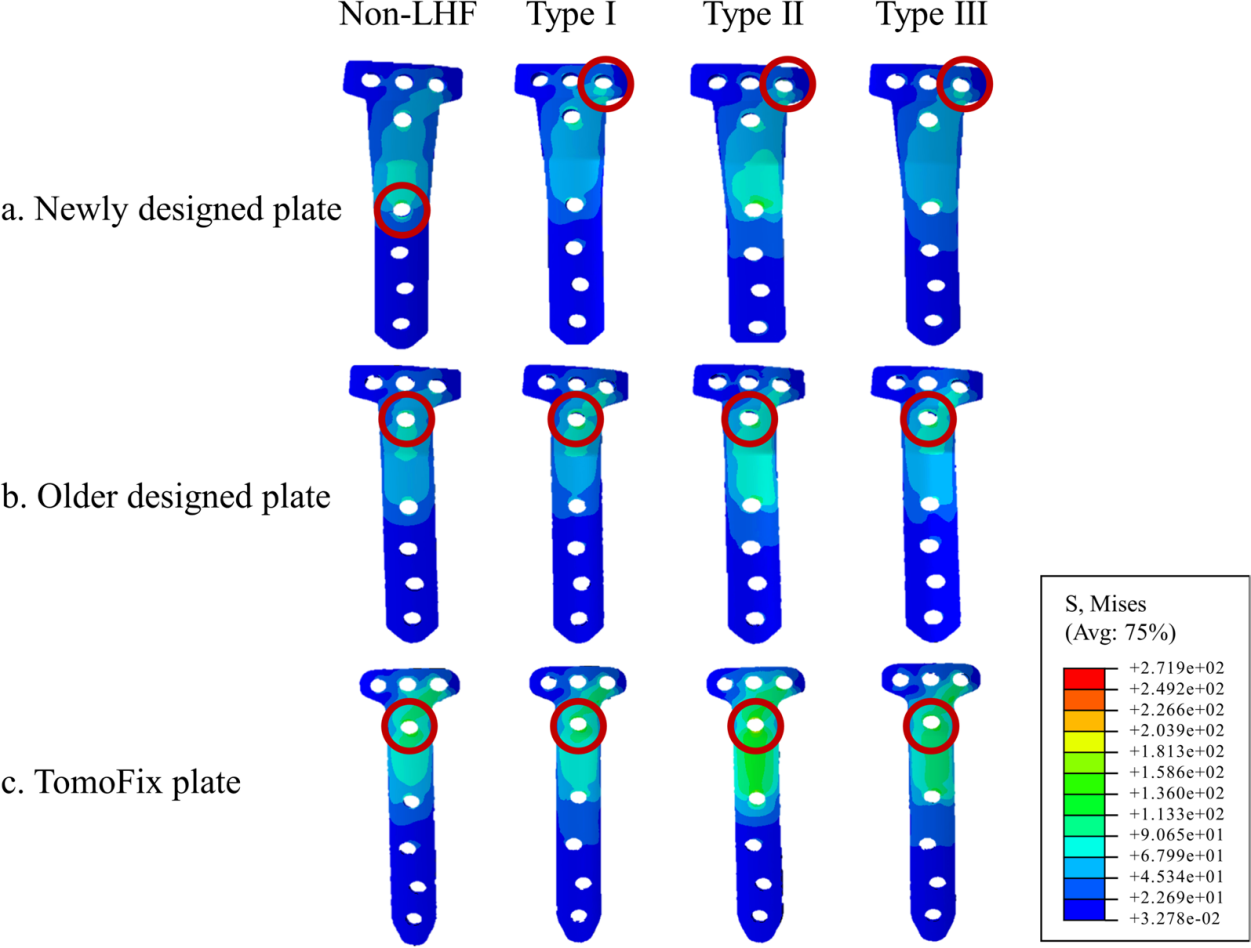
Finite element analysis showing Peak von Mises stress distribution around the locking plates **a** New OhtoFix; **b** Old OhtoFix; **c** TomoFix plates under different conditions such as without lateral hinge fracture, and Type I, Type II, and Type III lateral hinge fractures

Even in the presence of LHF, the peak von Mises stress around the D-hole was lower in the newly designed locking plate than in the old OhtoFix and TomoFix plates. The peak von Mises stress around the D-hole was highest in all three plates with a type II LHF, measuring 218.1 MPa in the newly designed plate, 310.4 MPa in the old OhtoFix plate, and 267.0 MPa in the TomoFix plate (Table 1, Fig. 6a). However, the peak stress across the entire plate was reduced in the newly designed plate compared with the old OhtoFix plate, but it remained higher than that of the TomoFix plate (Table 2, Fig. 6b). Additionally, in both the old OhtoFix and TomoFix plates the peak stress remained at the D-hole, regardless of the presence of LHF; however, the peak stress shifted to the C-hole in the newly designed plate (Fig. 7). In cases of type II LHF, the peak von Mises stress measured 271.9 MPa at the C-hole in the newly designed plate, whereas, in the old OhtoFix plate and TomoFix plate, the peak stress remained at the D-hole and measured 310.4 MPa, and 267.0 MPa, respectively.

## Discussion

Stable fixation around the osteotomy site is crucial to prevent complications in OWHTO [1]. Although several locking plates have been developed to enhance stability, there is no consensus on which structural design offers biomechanical superiority [2, 5–7]. Vulnerability in the locking plates, particularly around the D-hole has been reported, with stress in this area leading to plate breakage (especially in the presence of LHF) [9, 12, 16, 17]. To overcome this limitation, a newly designed locking plate has been developed [19]. The principal finding of our study was that the newly designed plate effectively reduced peak stress around the D-hole compared with both the old OhtoFix and TomoFix plates, consistently demonstrating the lowest peak stress regardless of the presence of LHF. However, it is important to note that the peak stress across the entire plate in the newly designed plate did not show improvement compared with the TomoFix plate. Additionally, the location of peak stress was shifted to the C-hole in the presence of LHF.

The study results confirmed our hypothesis that the newly designed locking plate reduced peak stress around the D-hole. However, while the modification successfully lowered stress in this specific area, it did not result in a significant improvement in the peak stress distribution across the entire plate. Previous biomechanical studies using TomoFix plate have reported that peak stress was found around D-hole in OWHTO [12, 16]. Similar results were observed in our study in the TomoFix and old OhtoFix plates. This was likely due to the bending of the plate to achieve anatomical fitting in the proximal tibia which creased a stress concentration around the D-hole making this area structurally more vulnerable [33]. Elevated peak stress was closely associated with the increased risk of plate failure which led manufacturers to develop locking plates designed to minimize stress concentrations [34, 35]. The stiffness of a plate, defined as its ability to resist deformation, is influenced by the properties of the material (known as Young’s modulus) and the structural design of the plate [36, 37]. In response to increased stress around the D-hole, the OhtoFix plate was re-designed with increased thickness and width of the plate [19]. Our study found that these structural modifications significantly reduced stress around the D-hole. However, despite these modifications, peak stress across the entire plate did not significantly improve compared with the old OhtoFix plate and TomoFix plate. Furthermore, the peak stress location was shifted to the 1-hole located in the distal portion of the area where the plate was bent. Therefore, when an orthopedic surgeon conducts OWHTO with the newly designed plate special attention should be given to the area around the 1-hole, where the peak stress occurred.

The study results confirmed our hypothesis that the newly designed locking plate endured its peak stress around the D-hole even when LHF occurred. However, peak stress across the entire plate did not surpass that of the TomoFix plate. One study of 12 case series demonstrated plate breakage around the D-hole in OWHTO associated with the presence of LHF [9]. Biomechanical studies have shown that when LHF occurred, peak stress around the D-hole increased and extended across the region from the D-hole to the proximal hole of the distal screws [12, 16]. Our study results aligned with that of the previous studies [12, 13, 16]. We found that the modifications made to the plate effectively reduced stress around the D-hole compared to the old OhtoFix plate and TomoFix plates potentially decreasing the risk of D-hole plate breakage. Although peak stress across the entire plate improved compared to the old OhtoFix plate, it did not surpass the performance of the TomoFix plate. Additionally, the location of peak stress shifted to the C-hole in LHF. This could be attributed to the 5-degree posterior slope in the proximal portion of the plate which resulted in greater stress being applied to the posterior aspect rather than the anterior aspect of the plate during loading [5]. Additionally, in LHF, the load distribution on the plate becomes compromised leading to increased stress around the C-hole rather than the reinforced D-hole. Therefore, our study results suggested that when OWHTO is conducted with the newly designed plate, the orthopedic surgeon should be mindful of the potential plate breakage at the C-hole, particularly in LHF.

This study has several limitations. First, our study was based on a simulated model rather than a clinical study. However, although it was possible to conduct a randomized controlled trial study using the newly designed locking plate, ethical concerns prevented the iatrogenic induction of LHF. Alternatively, a retrospective study could be conducted but it would not provide definitive information about the stress distribution characteristics of the newly designed plate compared to the other plate. Therefore, our study provided a unique advantage by directly assessing the biomechanical properties. Second, our study was performed under a single loading condition rather than a cyclic loading condition. It was possible that cyclic loading, which accounted for fatigue failure, could yield different results. However, it is worth noting that the minimum peak stress observed in cases of plate breakage associated with LHF was 241.1 MPa, as seen in the TomoFix plate with LHF Type I. In the newly designed locking plate, the minimum peak stress observed in LHF was 254.5 MPa which was higher than the aforementioned stress. Therefore, it was likely that the redistribution of peak stress in the newly designed locking plate could lead to plate breakage at the C-hole where peak stress was concentrated.

## Conclusions

The newly designed locking plate reduced stress around the D-hole confirming its potential for decreasing the risk of plate breakage in OWHTO. However, the modifications did not significantly enhance the overall stress distribution across the entire plate, and peak stress also did not surpass that of the TomoFix plate. Our study revealed that peak stress shifted to the C-hole in LHF suggesting a new area of vulnerability. These findings highlight the need for orthopedic surgeons to pay special attention to potential concentration at the C-hole in LHF when using the newly designed locking plate.

## Data Availability

The data supporting the findings of this study are available from the corresponding author upon reasonable request for research purposes.

## List of abbreviations

OWHTO: Opening Wedge High Tibial Osteotomy
LHF: Lateral Hinge Fracture
PVMS: Peak von Mises Stress
CT: Computed Tomography
MPa: Megapascal
Ti6Al5V ELI: Titanium Alloy
IRB: Institutional Review Board
